# Protocol for a randomised controlled trial examining the impact of a web-based personally controlled health management system on the uptake of influenza vaccination rates

**DOI:** 10.1186/1472-6963-12-86

**Published:** 2012-04-02

**Authors:** Annie Y S Lau, Vitali Sintchenko, Jacinta Crimmins, Farah Magrabi, Blanca Gallego, Enrico Coiera

**Affiliations:** 1 Centre for Health Informatics, Australian Institute of Health Innovation, University of New South Wales, Sydney, Australia; 2 Sydney Medical School, University of Sydney, Sydney, Australia; 3 Centre for Infectious Diseases and Microbiology, Institute of Clinical Pathology and Medical Research, Westmead Hospital, Sydney, Australia; 4 University Health Service, University of New South Wales, Sydney, Australia

**Keywords:** Randomised controlled trial, Influenza, Health promotion, Vaccination, Personally controlled health record, Self-management, Internet

## Abstract

**Background:**

Online social networking and personally controlled health management systems (PCHMS) offer a new opportunity for developing innovative interventions to prevent diseases of public health concern (e.g., influenza) but there are few comparative studies about patterns of use and impact of these systems.

**Methods/Design:**

A 2010 CONSORT-compliant randomised controlled trial with a two-group parallel design will assess the efficacy of a web-based PCHMS called *Healthy.me *in facilitating the uptake of influenza vaccine amongst university students and staff. Eligible participants are randomised either to obtain access to *Healthy.me *or a 6-month waitlist. Participants complete pre-study, post-study and monthly surveys about their health and utilisation of health services. A post-study clinical audit will be conducted to validate self-reports about influenza vaccination and visits to the university health service due to influenza-like illness (ILI) amongst a subset of participants. 600 participants older than 18 years with monthly access to the Internet and email will be recruited. Participants who (i) discontinue the online registration process; (ii) report obtaining an influenza vaccination in 2010 before the commencement of the study; or (iii) report being influenced by other participants to undertake influenza vaccination will be excluded from analysis. The primary outcome measure is the number of participants obtaining influenza vaccination during the study. Secondary outcome measures include: number of participants (i) experiencing ILI symptoms, (ii) absent from or experiencing impairment in work or study due to ILI symptoms, (iii) using health services or medications due to ILI symptoms; (iv) expressing positive or negative attitudes or experiences towards influenza vaccination, via their reasons of receiving (or not receiving) influenza vaccine; and (v) their patterns of usage of *Healthy.me *(e.g., frequency and timing of hits, duration of access, uptake of specific functions).

**Discussion:**

This study will provide new insights about the utility of online social networking and PCHMS for public health and health promotion. It will help to assess whether a web-based PCHMS, with connectivity to a health service provider, containing information and self-management tools, can improve the uptake of preventive health services amongst university students and staff.

**Trial registration:**

ACTRN12610000386033 (Australian New Zealand Clinical Trials Registry)

## Background

Influenza is an important contributor to population morbidity and mortality. Despite the unpredictable nature of influenza severity and spread on a population scale, understanding the behaviour of individual patients during seasons of respiratory disease has become essential for the planning and execution of successful public health interventions. However, current knowledge is based largely on observational studies, and randomised controlled experiments that test different public health interventions are urgently needed [1].

The pandemic of Influenza A H1N1 (Swine Flu) in 2009 and 2010 provided a major challenge to health services around the world. Similar to previous pandemics, it has led to significant aberrations in consumer behaviour, such as the stockpiling of goods, the victimisation of specific population groups, the cancellation of travel and the boycotting of particular foods (e.g., pork). Furthermore, large regional differences in risk estimation and risk perception have potentially affected the uptake of vaccination and other public health measures [2]. The pandemic prompted search for novel and innovative containment interventions as many traditional public health measures failed to control the spread of influenza infection. In particular, increasing the uptake of vaccination and ensuring compliance with containment policies have become a major challenge for public health and health authorities. The clinical picture in severe cases of pandemic (H1N1) 2009 influenza was also markedly different from the disease pattern seen during epidemics of seasonal influenza, in that many of those affected were previously healthy young adults with complex social interaction patterns. This group represents users of healthcare services who are most likely to be receptive to personal health records (PHRs), offering an opportunity to study and potentially modify healthcare information seeking and service utilization behaviour in young adults.

Online social networking and personally controlled health management systems (PCHMS) offer a new opportunity for developing innovative interventions to prevent diseases of public health concern [3]. Health promotion programs based on PCHMS can be used in a variety of settings targeting a large range of health issues, including the prevention of influenza [1]. Consumers are increasingly using such online systems to inform health decisions and manage their health. The uptake of PCHMS is likely to vary in different social groups under different conditions and within different contexts [4], but few comparative studies are available about patterns of use and impact of these systems.

It is thus important to ensure that design of studies to test the impact of online PCHMS adequately reflects the increased complexity of such interventions. This randomised controlled trial (RCT) will make a specific and significant contribution to our understanding on the efficacy of using a web-based PCHMS called *Healthy.me*, developed at the University of New South Wales, in improving the uptake of influenza vaccination in a university setting.

### Study aims and hypotheses

Specific hypotheses to be tested in this study are that:

1. Consumers using a PCHMS are more likely to comply with public health recommendations, as measured by their rates of seeking and obtaining influenza vaccination;

2. PCHMS that provides online facilities to schedule encounters with a health service provider will increase the utilisation rate of those services.

## Methods/Design

### Study design

**Figure 1:**
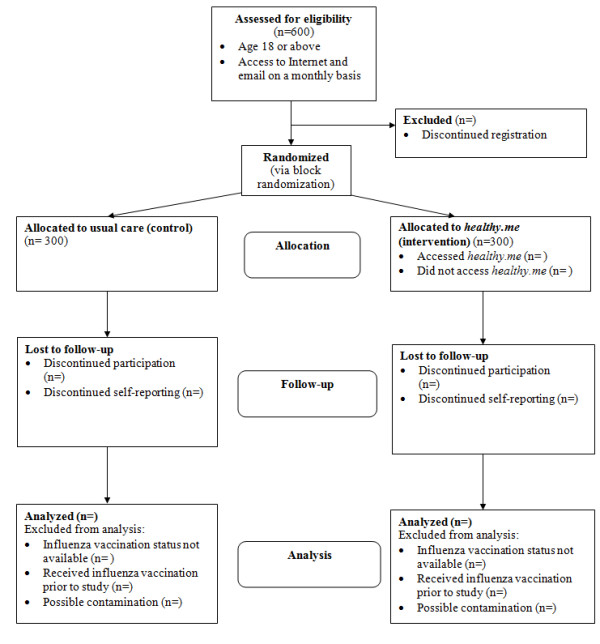
**Overview of RCT process**.

A randomised controlled trial with a two-group parallel design (with intervention allocation ratio 1:1) is used to evaluate the efficacy of *Healthy.me*, reported in accordance to the 2010 CONSORT statements (Figure 1) [5]:

• Participants randomised to the *intervention *group will have immediate access to *Healthy.me *from the date they are recruited in addition to health care of usual standard.

• Participants randomised to the *control *group will receive usual care. They will be allocated to a waiting list and delayed to use *Healthy.me *by approximately six months.

### Participants and setting

Table 1 outlines participant inclusion and exclusion criteria. The study will recruit students and staff members at UNSW who are fluent in English. Written material advertising the study outlines participant inclusion criteria, which are self-explanatory (i.e. age ≥ 18 and monthly access to Internet and email). Exclusion criteria are applied post-study. Hence, participants who self-identify as meeting inclusion criteria proceed to first providing informed consent before commencing the pre-study survey.

**Table 1:** Eligibility criteria for participants

Inclusion criteria	Exclusion criteria
1. Aged 18 or above.	1. Participants who did not complete registration process [excluded before randomization].

1. Access to the Internet, and email at least on a monthly basis.	2. Participants who self-reported having obtained an influenza vaccination in 2010 prior to enrolment in the study [excluded from analysis].

	3. Participants who self-reported to be influenced by other participants during the study to obtain (or not obtain) influenza vaccination [excluded from analysis].

Participants engage in this trial online in their own space at their own time. All participants are required to complete online (i) a 5-10 minute pre-study survey; (ii) a monthly one-minute survey to collect their health symptoms and health service use; and (iii) a 5-10 minute post-study survey. Participants allocated to the *Healthy.me *group are also required to complete a five-minute mandatory online tutorial about system before commencing the study. Those who complete all study surveys will enter into a draw for $500 prize at study completion.

#### Recruitment strategy

Five methods will be used to invite students and staff at UNSW to participate in the study (to avoid influencing consumer behaviour, it is not explicit to infer from advertising material that the study is about influenza or influenza vaccination):

1. Weekly and monthly advertisements circulated in online and print newsletters and magazines to staff and students.

2. Online announcements circulated for students and staff on UNSW home page, myUNSW home page, UNSW elearning portals and UNSW career websites.

3. Online annoucements circulated on Walls and discussion forums of Facebook accounts associated with UNSW student clubs and societies on campus.

4. Email invitations sent to Heads of Schools, Heads of UNSW departments, Heads of residential colleges, and delegates of UNSW student clubs and societies for dissemination to staff and student mailing lists.

5. Paper posters and leaflets placed on billboards across the university campus.

#### Ethical concerns and consent

Ethics approval for this study was obtained from the UNSW Human Research Ethics Committee. Participants provide written consent online; the revocation of consent form is also available online.

### Intervention and control

Volunteers responding to the invitation are required to register online by providing consent, and completing the pre-study survey and the tutorial about *Healthy.me*.

The control and intervention arms of the trial run concurrently, meaning that the randomly allocated participants of the trial are exposed to the same influenza season and the same background of public health messaging. The only difference between the 2 arms is exposure to the intervention. Whilst there are clear annual differences in flu and vaccination rates, our study should detect if in a given year we are able to change consumer behaviour. It is likely that in different years the degree of effect will differ, and only long term and longitudinal studies would help us understand the underlying trend. Such studies would be contemplated after strong evidence from this study that the intervention in principle is capable of impacting behaviour in a single season.

#### Description of intervention

**Figure 2:**
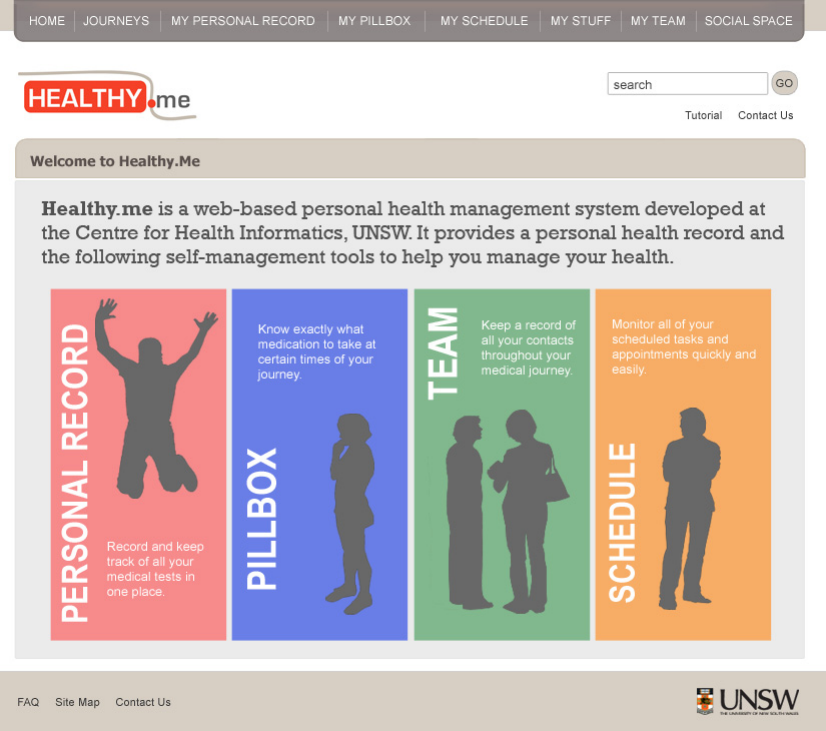
**Features of *Healthy.me *(^© ^University of New South Wales, 2009-2012)**.

The intervention in this trial is a web-based PCHMS called *Healthy.me*, which consists of the following features (Figure 2):

1. Personal Record: Allows for self-recording of medical test results and health measurements (not available to participants in this study).

2. Pillbox: Allows for self-recording of current medications and medication adherence.

3. Schedule, to-do list and reminders: An online schedule to self-record and keep track of health-related appointments, to-do items, which sends email reminders.

4. Team: A feature that allows the self-recording of clinical and non-clinical personnel looking after one's health.

5. Journeys: Consumer-specific care pathways that provide knowledge in an actionable way. A feature that describes the different stages in the management of health conditions that can be used to personalise other PHR sections in the system, and provides advice on what to expect and how to prepare for each stage. (Participants in this study had access to an influenza vaccine journey).

6. Social features for this study included: (i) a profile for each member to store and customise their personal information, (ii) the ability to send and receive messages with other members on *Healthy.me*.

Studies suggest that by addressing both knowledge-based (e.g. lack of awareness) and system-based (e.g. inconvenience) barriers in accessing health services, patients and consumers are more likely to uptake preventative health measures, such as influenza vaccination [6]. Our PCHMS is designed to address both knowledge-based and system-based barriers faced by consumers when accessing and utilising health services.

The PCHMS thus integrates a number of tools to address knowledge and system barriers that influence consumers' engagement with health services. Our PCHMS contains a PHR, as well as online social networking mechanisms that allow participants to interact with healthcare professionals at the service provider and other consumers, and additional tools to support consumer decision making and subsequent task management. In addition, it is integrated with consumer-specific care pathways (called "journeys") that provide knowledge in an actionable way. For example, at the point that a consumer encounters advice to seek influenza vaccination, they are immediately able to book an appointment with a medical practitioner, or set themselves a reminder.

The PCHMS was previously tested in a small trial with women undergoing in-vitro fertilisation (IVF), to demonstrate system usability and acceptance. Interview data from this trial provided evidence that the system positively impacted consumer advice-seeking behaviours [7].

**Figure 3:**
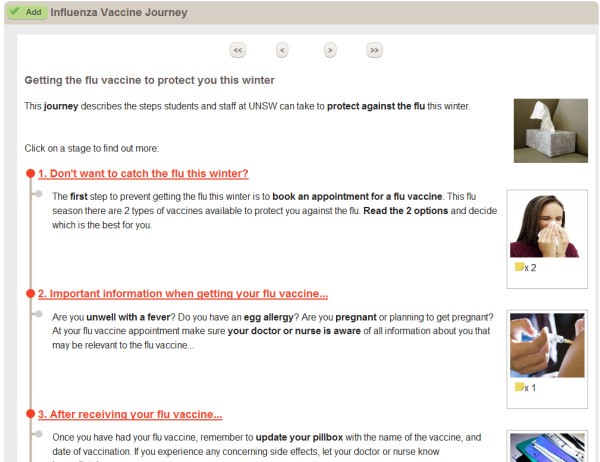
**Top page of *Healthy.me *influenza vaccine journey (^© ^University of New South Wales, 2009-2012)**.

The influenza vaccine journey in *Healthy.me *was iteratively developed and validated in consultation with the University Health Service (i.e. university's general practice) (Figure 3). It utilises government-endorsed evidence-based consumer education material that had been tested in the previous year to inform the UNSW community about seasonal influenza and pandemic H1N1 influenza [8-11].

#### Intervention group and exposure

**Figure 4:**
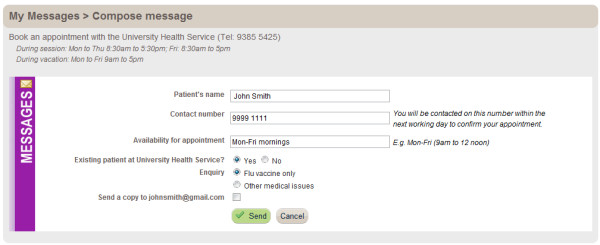
**Booking appointment with University Health Service on *Healthy.me *(^© ^University of New South Wales, 2009-2012)**.

The period of access to *Healthy.me *will vary depending on the date of participant registration. During the study *Healthy.me *will provide participants in the intervention group with information and forward email reminders about influenza, indications for vaccination, as well as an email link to the University Health Service for participants to book an appointment for influenza vaccination or other medical concerns, should they wish to use that service (Figure 4).

The intervention will not modify in any way the standard procedures of healthcare provision by the University Health Service. Only administrative staff at the practice would receive requests for appointments facilitated by *Healthy.me*; no clinical staff assessing patient outcomes and/or administrating influenza vaccination would receive these appointment requests.

A pilot was conducted in a controlled university setting with three men and three women of different ethnic and cultural backgrounds and age groups, representative of the university student and staff population. Substantive issues on system usability, journey content, surveys, study protocol and advertisement material were resolved before recruiting UNSW students and staff to participate in their normal setting.

#### Control

Participants in the control group will receive delayed access to *Healthy.me *by approximately 6 months.

### Sample size considerations

The sampling unit is the participant and the unit of analysis is also the participant. A conservative estimate of 600 participants with 300 in each group is needed to detect a 10% difference in influenza vaccination rate between the control group (15%) and the intervention group (25%). This estimate is calculated at 5% level of significance, 80% power (two-sided test), with an anticipated participant dropout rate around 10% [12].

The effect size estimate is based on a review assessing the effectiveness of patient reminder systems in improving immunization rates, which can range from 5 to 20% [13]. The baseline rate estimate of influenza vaccination was informed by our audit conducted at the University Health Service, showing 16% of patients at the practice received FluVax or PanVax between 5/3/2009 to 5/11/2009. This estimate is also supported by literature reporting 18 to 30% of university students and healthy adults (18 to 49 years old) obtaining influenza vaccination in a year [14,15].

### Outcome measures

Table 2 outlines the primary and secondary outcome measures in this study. Participants in control and intervention groups who are analysed for primary outcome will have completed the post-study survey and/or provided influenza vaccination status by de-identified clinical audit. Participants in intervention group who had the opportunity to use *Healthy.me *but did not do so will be included in the primary analysis. Participants who met the exclusion criteria or whose influenza vaccination status was unavailable at study completion will be excluded.

**Table 2:** Primary and secondary outcome measures collected at different time points

Outcome measure	Measurement timepoints & methods
*Primary outcome*	
Proportion of participants obtaining influenza vaccination during the study	• Study completion^1 ^(via self-reports and clinical audit)
*Secondary outcome*	
Proportion of participants visiting the University Health Service during the study	• Study completion^1 ^(via self-reports and clinical audit)
*Ancillary outcomes*	
Proportion of participants experiencing symptoms of influenza-like illness ^2 ^(ILI) during the study	• Monthly from study commencement in May to October 2010 (via self-reports)
Proportion of participants using medications or remedy due to ILI symptoms ^2^	• Monthly from study commencement in May to October 2010 (via self-reports)
Proportion of participants visiting a healthcare professional due to ILI symptoms ^2^	• Monthly from study commencement in May to October 2010 (via self-reports)
Proportion of participants experiencing impairment in work or study due to ILI symptoms ^2^	• Monthly from study commencement in May to October 2010 (via self-reports)
Number of days absent from work or study due to ILI symptoms (per participant)	• Monthly from study commencement in May to October 2010 (via self-reports)
Reasons for receiving (or not receiving) influenza vaccine	• Study completion^1 ^(via self-reports)
Patterns of usage and feedback of PCHMS	• Study completion^1 ^(via automatic system logs, data entered by participants into PCHMS, and self-reports)

^1^Estimated end of average respiratory disease and influenza season in Southern Hemisphere (i.e. October 2010, six months from study commencement)

^2^Defined by case definitions of influenza (fever with cough or a sore throat) issued by NSW Health and Centers for Disease Control and Prevention (CDC) as of 26 March 2010

#### Influenza like-illness (ILI) case definition

ILI symptoms are based upon case definitions of influenza issued by NSW Health and Centers for Disease Control and Prevention (CDC) as of 26 March 2010 [16,17]. Febrile upper respiratory tract illnesses occurring during the peak influenza period was identified as the most specific clinical case definition expected to have the highest positive predictive value for true influenza [18].

### Data collection

Four methods will be used to collect data about participants during the study:

*Surveys*: Questions are informed by studies tracking ILI symptoms in the Australian community [19,20], and studies investigating influenza experiences and attitudes amongst university students and healthy working adults [14,21-26]. The following surveys will be conducted:

• Pre-study survey to obtain participant demographics and health status at study registration.

• Monthly one-minute follow-up surveys to obtain participant self-reported symptoms of ILI, use of health services, and impact on work and study due to ILI symptoms throughout the study.

• Post-study survey to obtain participant influenza vaccination status. For those who receive *Healthy.me *intervention, they will be asked in addition for their perceived usefulness of *Healthy.me*.

*System logs*: During the study, participant actions on *Healthy.me *will be unobtrusively logged.

*Personal health data*: Data entered by participants into *Healthy.me *about their health (e.g., medications used, scheduled tasks and appointments, members in healthcare team).

*Clinical audit*: This will be conducted post-study for patients at the University Health Service (UHS), to validate self-reports of vaccination and visits to a healthcare professional related to ILI symptoms for a subset of participants before and during the study. Clinical audits will be conducted on site at the UHS, linking UHS patient records with our survey data using exact matches on participants' surname, date of birth, and an approximate match on first-name. Extracted records will be replaced with a de-identified ID, and all names will be removed prior to analysis.

### Analysis plan

Statistical significance is defined a priori as a P value of less than 0.05 (determined using a two-tailed test). Data will be collected by online survey software KeySurvey [27] and analysed using PASW Statistics 18 [28].

#### Baseline comparisons

Comparisons of baseline variables between PCHMS and waitlist groups will be conducted using Student's t-tests for continuous variables and χ^2 ^tests for categorical variables, to assess whether randomisation was performed properly. Adjustment for baseline characteristics is planned to correct for possible imbalance between the randomised groups and to provide a stratified estimate of the effect of intervention if applied [29,30].

#### Primary analysis

Differences in proportions of participants obtaining influenza vaccination during the study will be compared between waitlist and PCHMS groups. All intervention recipients who had the opportunity to use the PCHMS but did not do so will be included in the primary analysis. Differences in participant proportions between waitlist and PCHMS groups will be analysed using χ^2 ^test. Proportions will be reported with 95% confidence intervals.

Adjustments for baseline characteristics and potential confounders will be made through the use of sequential logistic regression [31]. Baseline characteristics (Table 1), and factors that may affect influenza vaccination collected at post study (i.e. contact with children during study, past history of influenza vaccine) will be entered at Step 1 of the regression; and group allocation (PCHMS vs. waitlist) will be entered at Step 2.

#### Secondary and ancillary analyses

Differences in proportions of participants between different groups (e.g. waiting list vs. PCHMS; used PCHMS once vs. used PCHMS more than once) will be examined using χ^2 ^test for each of the following activities experienced at least once during the study: i) visited the University Health Service (or a healthcare professional); ii) used medications or remedy; and iii) experienced performance impairment. Differences in average number of days of absence per participant will be compared between waitlist and PCHMS groups using Student's t-test. Reasons for receiving (or not receiving) influenza vaccine and impact of ILI symptoms will be reported using descriptive statistics.

### Study procedure

Table 3 summarizes participant procedures in the study. The duration of the study is expected to be six months from May 2010 to October 2010.

**Table 3:** Stages of study procedure

Stage of study	Procedure
Online registration	- Participant registration, study consent and Healthy.me tutorial (self-completed online)

Baseline	- Pre-study survey (self-completed online)
	- Data from de-identified clinical audit from previous 12 months: electronic extraction (collected post-study)

Follow-up procedures	- Monthly one-minute surveys (self-completed online)
	- Post-study survey (self-completed online)
	- Patterns of *Healthy.me *use (computer logs and data entered by participants)
	- De-identified clinical audit at study completion: electronic extraction (collected post-study)

Email will be the primary channel to communicate with participants for study information and reminders about survey completion. From the time participants are recruited until study completion, all participants (control & intervention) will receive an email in the first week of each month to complete a one-minute survey about their health in the past month. At study completion, all participants will receive an email asking them to complete a post-study survey. Two follow-up emails five days apart from each other will be sent to remind those who have not completed each survey to ensure the completeness of data collection.

#### Randomisation and allocation concealment

After consent each participant is randomly allocated to the intervention or control group, facilitated by a computerised centralised allocation process which forms part of the online registration procedure. Eligible healthcare consumers are randomly assigned to the intervention or control group using a computer generated random number sequence in randomly assigned blocks (block sizes 2, 4 and 8) with intervention allocation ratio of 1:1 [32].

The randomisation sequence generation, participant registration, and group allocation processes are computerised online and do not involve human interventions from the investigators. The block randomisation sequence is pre-generated by a member external to the research team using a computerised random-number generator before commencing participant recruitment. As each participant completes the online registration procedure, he/she receives the next consecutive allocation in the sequence, which automatically assigns the consumer to the intervention or control group.

#### Blinding and assessment

Since *Healthy.me *is a behavioural intervention it is not possible to completely blind participants to the intervention. The group allocation is revealed to participants only after obtaining their consent to participate in the study and completion of the pre-study survey. However, investigators and clinicians involved in the study are blinded to group allocation. To minimise contamination of control participants who might interact with participants who are part of the intervention group, participants in the intervention group are asked not to share their *Healthy.me *access details with other people. Further, participants who self-reported to have been influenced by others participating in the study to obtain (or not obtain) influenza vaccination will be excluded from analysis. To assess the success of blinding, administrative staff at the University Health Service will be consulted at study completion to confirm whether clinicians involved in assessing patient outcomes and/or administrating influenza vaccination are able to distinguish patients who booked an appointment using *Healthy.me*.

## Discussion

Health promotion and surveillance have received unprecedented recognition because of newly emerging and re-emerging infectious diseases with epidemic potential, new cycles of pandemics, and the threats of bioterrorism. Influenza and influenza-like illness (ILI) are important contributors to population and individual morbidity and mortality. The recent influenza pandemic with novel H1N1 has highlighted the need for a better understanding of patients' healthcare seeking behaviour and perceptions and attitudes towards vaccination. Findings of our study will help to facilitate health promotion and surveillance in technology savvy populations as well as to enhance healthcare professionals' and governments' capacity to predict and prepare for the subsequent epidemics and pandemics.

The study will further explore the role of clinical illness case definitions for influenza as methods used to define influenza seasons have varied substantially. These differences often result in differing levels of sensitivity, specificity and positive predictive value for the case definitions used. Furthermore, these differences affect the estimation of influenza vaccine effectiveness, safety and cost benefit analyses in healthy working adults [24,33].

## Limitations

There are several potential limitations in this study:

• *Number of eligible participants*: Recruitment may be affected by an overlap between the start of respiratory season in Australia and the student examination period, potentially affecting student and staff availability and interest to participate in research studies. The number of participants meeting exclusion criteria at study completion might be significant because staff members are eligible for free influenza vaccination offered by UNSW one week prior to study commencement. As free vaccination would be a confounder if included in this trial, we explicitly ran our campaign independently of the university program.

• *Self-reports of influenza vaccination and health service utilisation*: Since clinical audits will only be conducted at the University Health Service, and not other GP practices, the study is dependent on participants' self-reporting of their influenza vaccination and health service utilisation. Self report has shown to be acceptably accurate in numerous studies examining days of absence [34], influenza symptoms, and vaccination status for diverse patient cohorts [20,33,35-38]. We will minimise the risk of recall bias by conducting short one-minute monthly follow-up surveys on the first week of each month.

• *Representative of general healthcare consumers*: The study may be more appealing to consumers who are interested or literate in computers, the Internet, or health-related topics. These participants may be more enthusiastic about health and the Internet than the general health consumer population. In addition, participants from a university setting are more likely to be open and positive to new research ideas and willing to participate in research studies. Further, generalisability of findings could be limited by recruiting participants from one university.

• *Social influence and access to Healthy.me*: The social networks of individual participants may span the intervention and control arms of this study, and see intervention subjects un-blind control subjects with information obtained from the intervention. As this is a pragmatic trial in the real world, such effects cannot be controlled for. Indeed participants' social networks are likely to influence access to Healthy.me if the intervention was in routine use. We will attempt to measure this effect by asking participants in the control arm if they have been in contact with intervention arm subjects. If a significant group is identified, we will conduct separate analyses to estimate the impact of any such effect on overall trial results.

• *Baseline comparisons*: As this is a pragmatic trial of a multifaceted intervention in a complex environment, it is possible that baseline variables associated with subjects might also influence the outcome. For example, having a prior history of obtaining influenza vaccination may predict future vaccination rates, independently of any additional intervention. We will identify potential baseline variables that might influence outcome, including gender, and test for unequal distribution of these variables in the intervention and control populations. Should there be a significant difference in their distribution due to chance bias, these will be fitted in an analysis of covariance to model their impact on any observed differences between the two arms [39].

### Concluding remarks

Our design of this randomised controlled trial (RCT) focuses on the comparison of outcomes that unequivocally reflect a change in consumer behaviour, and takes into account the complexity of intervention. Results of this study will offer new insights about the utility of online social networking for public health and health promotion. Our findings will provide specific answers to whether using a web-based PHCMS, with connectivity to a health service provider, containing information and self-management tools that facilitate consumers to act on a decision, will improve the uptake of preventive health services amongst students and staff in a university setting.

## Competing interests

The university and some of the researchers involved in this project could in the future benefit from any commercialisation of *Healthy.me *or its technologies, but no such commercial plans have been developed.

## Authors' contributions

AL and EC conceptualised and designed the study. AL and VS wrote the first draft of the manuscript. All authors contributed to study design (or analysis), subsequent drafts of the manuscript, and have read and approved the final draft for submission.

## Pre-publication history

The pre-publication history for this paper can be accessed here:

http://www.biomedcentral.com/1472-6963/12/86/prepub
